# Bone mineral density is not associated with incident high-intensity back pain: a 10-year cohort study in men

**DOI:** 10.1093/jbmrpl/ziae076

**Published:** 2024-06-10

**Authors:** Mahnuma M Estee, YuanYuan Wang, Stephane Heritier, Donna M Urquhart, Flavia M Cicuttini, Mark A Kotowicz, Kara B Anderson, Sharon L Brennan-Olsen, Julie A Pasco, Anita E Wluka

**Affiliations:** School of Public Health and Preventive Medicine, Monash University, Melbourne, Victoria 3004, Australia; School of Public Health and Preventive Medicine, Monash University, Melbourne, Victoria 3004, Australia; School of Public Health and Preventive Medicine, Monash University, Melbourne, Victoria 3004, Australia; School of Public Health and Preventive Medicine, Monash University, Melbourne, Victoria 3004, Australia; School of Public Health and Preventive Medicine, Monash University, Melbourne, Victoria 3004, Australia; Deakin University, IMPACT—Institute for Mental and Physical Health and Clinical Translation, Geelong, Victoria, 3220, Australia; Department of Medicine–Western Health, The University of Melbourne, St Albans, Victoria, 3021, Australia; University Hospital Geelong, Barwon Health, Geelong Victoria, 3220, Australia; Deakin University, IMPACT—Institute for Mental and Physical Health and Clinical Translation, Geelong, Victoria, 3220, Australia; Australian Institute for Musculoskeletal Sciences (AIMSS), Western Health and University of Melbourne, St Albans, Victoria, 3021, Australia; Deakin University, School of Health and Social Development, Geelong, Victoria, 3220, Australia; School of Public Health and Preventive Medicine, Monash University, Melbourne, Victoria 3004, Australia; Deakin University, IMPACT—Institute for Mental and Physical Health and Clinical Translation, Geelong, Victoria, 3220, Australia; Department of Medicine–Western Health, The University of Melbourne, St Albans, Victoria, 3021, Australia; School of Public Health and Preventive Medicine, Monash University, Melbourne, Victoria 3004, Australia

**Keywords:** back pain, disability, bone mineral density, osteoporosis, men

## Abstract

Although patients believe that osteoporosis is a painful condition, health professionals assume it is painless unless a fracture occurs. The association between BMD and back pain has not been examined longitudinally in community-based adults in an unbiased population using gold-standard measures. This study aimed to examine the association between BMD and incident high-intensity back pain and/or high disability over 10 years in Australian men without high-intensity symptoms at baseline. Men with no high-intensity back pain and/or high disability attending the Geelong Osteoporosis Study at the 5-year visit (occurring between 2006–2010) (considered the baseline for the current study) were followed for 10 years (reassessed between 2016–2021). Back pain and disability were assessed using the Graded Chronic Pain Scale at both time points. At baseline, DXA was used to measure lumbar spine and total hip BMD and spinal artefacts. The relationships between BMD and incident high-intensity pain and/or high disability at follow-up were examined using binary logistic regression, adjusted for age, body mass index, depression, education, smoking, mobility, and spinal artefacts. A total of 679 participants had no to low-intensity pain and/or no to low disability at baseline. A total of 441 attended follow-up, providing back pain and disability data. Thirty-seven men developed high-intensity pain and/or high disability. No association of BMD at any site was seen with incident high-intensity pain and/or high disability. BMD was not associated with incident high-intensity pain or disability in community-based men. These data provide evidence to dispel the erroneous community-held belief that low BMD is related to back pain and disability.

## Introduction

Chronic back pain is the biggest contributor to the burden of disability worldwide.^1^ Back pain–related disability has increased by 52.7% since 1990.^1^ Approximately 28% of individuals with back pain have severe symptoms: these individuals account for nearly 80% of the back pain–related disability and utilize more than double the healthcare cost used by those with less severe back pain.^2^^,^^3^ Regardless of the cost, approximately 50% of people are dissatisfied with the management of their back pain.^4^ It is thus important to identify modifiable factors associated with the incidence of severe back pain and disability to enable effective prevention and to optimize health outcomes.

Patients’ beliefs related to back pain have been shown to affect back pain outcomes,^5^^,^^6^ including their understanding of its etiology.^5^^,^^6^ Several strategies have demonstrated that successfully shifting beliefs improves outcomes, suggesting a potential preventive strategy for back pain.^7-9^ Thus, it is important for healthcare professions to have evidence regarding other community misconceptions related to back pain to enable these to be dispelled.

Although healthcare providers believe osteoporosis is a painless condition in the absence of fracture,^10^ many in the community believe that osteoporosis is a painful condition.^11^^,^^12^ The data underlying this belief are unclear. Most previous studies examining this relationship have been cross-sectional,^13-23^ and have had conflicting results. This may be attributed to the use of unvalidated measures of back pain,^13-23^ poor measures of bone health (eg, historical fracture or BMD measurement at sites other than at lumbar spine or hip)^14^^,^^16^^,^^18^^,^^19^^,^^21^ or study performed in a potentially biased population.^13^^,^^15^^,^^16^^,^^23^ There has only been a single longitudinal study. This was population based and used lumbar BMD.^24^ However, it was performed in 36-year-old adults, who were assessed for back pain 6 years later. This high-quality study showed that low BMD was associated with increased risk of back pain in 36-year-old men, but not women. Thus, it is possible that BMD may be a modifiable risk factor for back pain.

No longitudinal study has been performed in a community-based population recruited without attention to BMD (exposure) or back pain (outcome), over a wide age range, to examine the association between BMD and incident back pain in men. It is feasible that low BMD may be associated with pain because preclinical data have shown that dysregulation of the bone remodeling due to mechanical stress, aging, or inflammation can also promote pain by increasing cytokine secretion or aberrant sensory innervation.^25^^,^^26^

Therefore, this study aimed to examine whether BMD measured using DXA was associated with the longitudinal incidence of high-intensity back pain and/or high disability in a population-based sample of Australian men with no or low-intensity pain and/or no or low disability at study baseline.

## Materials and methods

### Study population

**Figure 1:**
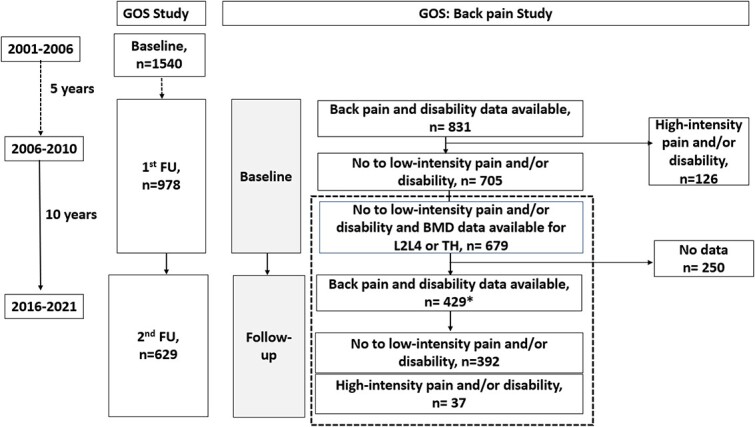
Participant flow in the Geelong Osteoporosis Study (GOS) and the current back pain study within the GOS. The dotted box indicates the current study. *431 participants provided pain data and 429 participants provided disability data at follow-up. Abbreviation: FU, follow-up.

The Geelong Osteoporosis Study (GOS) is a prospective, population-based cohort study of adults in Australia. Between 2001 and 2006, this study recruited a random age-stratified sample of 1540 men aged *>*20 years from the Barwon Statistical Division using the Australian electoral roll (Figure 1).^27^ A total of 978 men attended the GOS first follow-up visit (5 years, 2006–2010) and, at the time of writing, 629 had completed the GOS second follow-up visit (15 years, 2016–2021). The current study incorporates participants who provided back pain data at both the GOS first (2006–2010) and second (2016–2021) follow-up (Figure 1, dotted box). Hereafter, reference to the current study will be using baseline, referring to measures made at the GOS first follow-up (2006–2010), and follow-up, referring to the GOS second follow-up (2016–2021).

### Back pain and disability

The Graded Chronic Pain Scale (GCPS) questionnaire, a validated self-report instrument, was used to assess back pain and disability over the previous 6 months,^28^ at baseline and follow-up. The GCPS categorizes back pain, incorporating both the pain intensity and disability. This is because pain intensity alone may not be able to discriminate a higher level of pain severity.^28^ Participants are categorized into 5 groups (0 = no pain and disability, 1 = low-intensity pain and low disability, 2 = high-intensity pain and low disability, 4 and 5 = high-intensity pain and high disability).^28^ Those in grades 0 or 1 are referred to as the no- or low-symptoms group hereafter). All participants with high-intensity pain and/or disability (grades 2 through 4) are categorized together, and referred to as the high-symptoms group hereafter. Participants were also classified based on their pain-intensity score alone. Those with a pain-intensity score *>*50 were classified as developing high-intensity pain. Participants were also categorized on the basis of disability score alone. Those with a disability point score *>*3 at follow-up were classified as developing high disability.^28^ Subgroup analyses were performed to examine predictors of any incident pain or disability: (1) participants were classified as having either no pain and/or no disability (grade 0 at baseline and follow-up; referred to as “no symptoms group”) or having incident pain and/or disability (grade 0 at baseline and grade 1–4 at follow-up; referred to as low- or high-symptoms group) and (2) no deterioration from baseline low-intensity pain and/or disability (grade 1 at baseline and grade 0 or 1 at follow-up; referred to as no-deterioration group) versus deterioration with high-intensity pain and/or disability (grade 1 at baseline and grade 2, 3, or 4 at follow-up; referred to as deterioration group).

### BMD measurement

At baseline and follow-up, a GE-Lunar Prodigy DXA (Prodigy; GE Lunar, Madison, WI, USA) was used to measure BMD (g/cm^2^) at the lumbar spine (L2–L4), and total hip (TH) as these are the default regions of interest of this machine.^29^ A bone densitometry expert identified the presence of spinal artefacts (osteophytes, endplate sclerosis, fracture at lumbar region or thoracic region, scoliosis, aortic calcification, spondylosis, surgery, poor scan, and others) on posterior-antero lumbar spine and lumbar vertebral assessment scans at baseline.^30^ In the unusual situation where 1 of the vertebrae was an SD away from the others and/or noted to be badly deformed and an outlier, it was excluded after consultation with an experienced DXA technician or the senior doctor. T scores were calculated following the reference range for Australian men,^29^^,^^31^ enabling categorization of the population into normal BMD, osteopenia, and osteoporosis at each site using the World Health Organization (WHO) definition, which has been operationalized in men, and also using the lowest measure at any site.^32-35^

### Baseline measures

Age and BMI were calculated. Current smoking status was ascertained at baseline. Highest educational level was categorized into completed secondary school or lower vs above secondary school level.^36^ The Hospital Anxiety and Depression Scale (HADS-D) questionnaire was used to determine depressive features (total score, 0–21)^37^; higher scores indicate more severe depressive features (a cutoff *>*8 indicates depressive features).^37^ Mobility status was categorized as “high mobility” (very active and active) or “low mobility” (sedentary, limited, inactive and chair or bedridden, and bedfast).^38^

### Statistical analysis

Participants’ baseline characteristics (age, BMI, depression, education, smoking status, mobility), the percentage of participants with osteoporosis/osteopenia/normal BMD at any site or single site, and BMD at L2–L4, and TH with no or low-intensity back pain and/or no or low disability were compared with those developing high-intensity pain and/or high disability using independent-samples *t* tests or chi-square tests, as appropriate. Due to lower numbers, osteopenia and osteoporosis groups were combined for further analysis. Change in BMD was calculated for L2–L4, and TH by subtracting BMD measured at follow-up (2016–2021) from BMD at baseline (2006–2010). The percentage of BMD measures was calculated by multiplying the raw data by 100. Annual percentage change in BMD measures was calculated by dividing the percentage of BMD measures with initial BMD at 2006–2010 and years between 2 measurement times (time between 2006–2020 and 2016–2021). To assess associations between incident high-intensity pain and/or high disability and percentage measures of BMD at baseline or annual percentage change in BMD over 10 years, binary logistic regression was used. The analyses were adjusted for age, BMI, mobility, depression, education, smoking, artefacts, and time between measures. Artefacts were identified as being related to fracture, structural changes (endplate sclerosis, osteophytes, spondylitis, scoliosis, and surgery), and other artefacts (aortic calcification, poor scan, and others). Exploratory subgroup analysis was done based on age, depression, mobility, education, artefacts, and fracture and structural changes. Between-group interaction for adjusted analysis was calculated.

All statistical analyses were done using IBM SPSS Statistics for Windows (Version 27.0., Armonk, NY, USA) IBM Corp. A *p*-value less than .05 was considered significant.

### Ethical approval

Ethical approval from the Human Research Ethics Committees of Barwon Health (reference no. 00/56) and informed consent was obtained from all participants included in the study.^27^ A research collaboration agreement between Monash University and Barwon Health was obtained.

## Results

After excluding participants with high-intensity back pain or high disability and those who did not have BMD data at L2–L4 or TH areas at baseline (see Figure 1), 679 participants were followed up and pain and/or disability data were available for 429 (63.2%; age range, 24.1–87.7 years) at the return visit (Figure 1). Information regarding BMD at L2–L4 and TH was available for 428 and 422 participants, respectively, at baseline. Men lost to follow-up were significantly older (66.4 [18.5] vs 54.3 [14.0] years), more likely to have depressive features (HADS-D score, 3.3 [2.3] vs 2.5 [2.9]), had low mobility (83 [33.3%] vs 82 [19.2%]), did not complete secondary school (128 [52%] vs 169 [40.1%]), lower BMD at TH (1.05 [0.14] vs 1.08 [0.13]), and had higher artefacts (153 [62.4%] vs 200 [47.1%]) compared with those who completed the study. There were no significant differences between these 2 groups in terms of BMI and BMD at L2–L4 (Table S1).

The characteristics of men with no or low symptoms (*n* = 392, 91.4%) and men with high symptoms (*n* = 37, 8.6%) were compared (Table 1; mean differences [MDs] shown in Table S2). Men with high-intensity pain and/or high disability were more likely to have depressive features (MD, –1.4 [–2.4, –0.4]) and have low mobility (32.4%) compared with those who did not develop high-intensity pain and/or disability (17.9%, *p* = .04).

**Table 1:** Comparison of baseline characteristics of men with no or low-intensity pain and/or disability vs men with high-intensity pain and/or high disability at follow-up.

	**Pain and/or disability** ^**a**^			**Pain** ^**b**^			**Disability** ^**a**^		
	**No or low-intensity pain and/or disability (*n* = 392)**	**High-intensity pain and/or disability (*n* = 37)**	***p***	**No or low pain (*n* = 398)**	**High-intensity pain (*n* = 33)**	***p***	**No or low disability (*n* = 415)**	**High disability (*n* = 14)**	***p***
Age,^c^ y	54.1 (14.1)	56.3 (1.8)	.38	54.3 (14.1)	56.2 (13.7)	.44	54.3 (14.2)	55.9 (10.9)	.68
BMI,^c^ kg/m^2^	27.3 (3.8)	26.6 (4.2)	.33	27.2 (3.8)	26.9 (4.3)	.60	27.2 (3.8)	27.2 (4.8)	1.0
Depressive features^c^	2.4 (2.2)	3.7 (2.9)	.01	2.4 (2.2)	3.7 (2.9)	.02	2.4 (2.2)	4.6 (2.9)	.02
Did not complete secondary school^d^	153 (39.8%)	16 (43.2%)	.69	155 (39.7%)	16 (48.5%)	.33	164 (40.3%)	5 (35.7%)	.73
Low mobility^d^	70 (17.9%)	12 (32.4%)	.03	71 (17.9%)	11 (33.3%)	.03	77 (18.6%)	5 (35.7%)	.11
Any artefact^d^	180 (45.9%)	20 (55.6%)	.27	184 (46.2%)	18 (56.3%)	.28	189 (45.7%)	11 (78.6%)	.02
Fracture^d^	8 (2.1%)	1 (2.8%)	.77	8 (2%)	1 (3.1%)	.68	8 (1.9%)	1 (7.1%)	.14
Structural changes^d^^,^^e^	162 (41.6%)	19 (52.8%)	.20	166 (42%)	17 (53.1%)	.22	170 (41.4%)	11 (78.6%)	.01
Other artefacts^d^	30 (7.7%)	3 (8.3%)	.90	31 (7.8%)	2 (6.3%)	.75	31 (7.5%)	2 (14.3%)	.35
At any site (lumbar spine or total hip)									
Osteoporosis or osteopenia^d^	149 (38%)	15 (40.5%)	.76	151 (37.9%)	14 (42.4%)	.61	159 (38.3%)	5 (35.7%)	.84
Lumbar spine (*n* = 428)									
Osteoporosis^d^	2 (0.5%)	0 (0%)	.79	2 (0.5%)	0 (0%)	.86	2 (0.5%)	0 (0%)	.44
Osteopenia^d^	81 (20.7%)	6 (16.2%)		82 (20.7%)	6 (18.2%)		86 (20.8%)	1 (7.1%)	
Normal^d^	308 (78.8%)	31 (83.8%)		313 (78.8%)	27 (81.8%)		326 (78.7%)	13 (92.9%)	
BMD,^c^ g/cm^2^	1.28 (0.17)	1.32 (0.19)	.17	1.28 (0.17)	1.32 (0.19)	.21	1.28 (0.17)	1.35 (0.18)	.15
Total hip (*n* = 423)									
Osteoporosis^d^	3 (0.8%)	0 (0%)	.66	3 (0.8%)	0 (0%)	.60	3 (0.7%)	0 (0%)	.89
Osteopenia^d^	119 (30.7%)	13 (37.1%)		121 (30.8%)	12 (38.7%)		127 (31.1%)	5 (35.7%)	
Normal^d^	265 (68.5%)	22 (62.9%)		269 (68.4%)	19 (61.3%)		278 (68.1%)	9 (64.3%)	
BMD,^c^ g/cm^2^	1.08 (0.13)	1.07 (0.13)	.57	1.08 (0.13)	1.07 (0.14)	.66	1.08 (0.13)	1.06 (0.13)	.61

a Data available for 429 participants who provided pain and disability at both time points.

b Data available for 431 for participants who provided pain, but not disability data at both time points.

c Data presented as mean (SD); comparison *p*-value for independent *t* test.

d Data presented as *n* (%); comparison *p*-value for chi-square test.

e Structural changes includes endplate sclerosis, osteophytes, spondylitis, scoliosis, and surgery.

The characteristics of the study population, comparing men with no or low pain (*n* = 398, 92.3%) with men with high pain (*n* = 33, 7.7%) and those with no or low disability (*n* = 415, 96.7%) and those with high disability (*n* = 14, 3.3%) were also compared (Table 1; MDs shown in Table S2). Those with no or low-intensity pain (MD, –1.3 [–2.3, –0.5]) and those with no or low disability (MD, –2.2 [–3.9, –0.5]) had lower HADS-D scores compared with those with high-intensity pain or high disability. Further, a higher proportion of men who developed high-intensity pain had low mobility (33.3%) compared with those who did not (17.9%, *p* = .03). Men who developed high disability (78.6%) were more likely to have artefacts present on DXA than those who did not (46.0%, *p* = .02). No differences were detected for measures of BMD for men who did and did not develop high-intensity pain and/or disability.

The associations between BMD measures and developing high-intensity pain/high disability were examined (Table 2), adjusted for age, BMI, depression, mobility, education, smoking, and presence of spinal artefacts. No association was found between measures of BMD at any location and odds of developing high-intensity pain or high disability in univariable analysis or after adjustment. The presence of osteopenia/osteoporosis at a single site or at any site was not associated with developing high-intensity pain and/or high disability (Table 2).

**Table 2:** Association of measures of BMD with developing high-intensity pain or high pain-related disability.

	**High intensity pain and/or disability, OR (95% CI)**		**High-intensity pain, OR (95% CI)**		**High-intensity disability, OR (95% CI)**	
	**Unadjusted**	**Adjusted** ^**a**^	**Unadjusted**	**Adjusted** ^**a**^	**Unadjusted**	**Adjusted** ^**a**^
BMD (g/cm^2^)						
Lumbar spine	1.01 (0.99-1.03)	1.01 (0.99-1.03)	1.01 (0.99-1.03)	1.01 (0.99-1.03)	1.02 (0.99-1.05)	1.02 (0.99-1.05)
Total hip	0.99 (0.97-1.02)	1.00 (0.97-1.03)	0.99 (0.97-1.02)	1.00 (0.97-1.03)	0.99 (0.95-1.03)	0.99 (0.94-1.03)
Osteoporosis or osteopenia						
Any site	1.11 (0.56-2.21)	1.03 (0.49-2.17)	1.21 (0.59-2.48)	1.15 (0.53-2.50)	0.89 (0.29-2.72)	1.02 (0.31-3.38)
Lumbar spine	0.72 (0.29-1.78)	0.64 (0.24-1.68)	0.83 (0.33-2.07)	0.75 (0.28-1.98)	0.29 (0.04-2.21)	0.28 (0.03-2.33)
Total hip	1.28 (0.63-2.63)	1.14 (0.52-2.50)	1.37 (0.65-2.91)	1.29 (0.57-2.94)	1.19 (0.39-3.62)	1.27 (0.38-4.30)

Values are ORs (95% CI). The reference group is participants without osteoporosis or osteopenia any site, participants without osteoporosis or osteopenia at the lumbar spine, and participants without osteoporosis or osteopenia at the total hip accordingly. Abbreviation: OR, odds ratio.

a Adjusted for age, BMI, depression, mobility (low-mobility), education (did not complete secondary school or lower), smoking (not a current smoker), any artefact (present), and time between 2 measurements (between 2006–2010 and 2016–2021) (in years).

There was no association between BMD measures and developing any intensity pain and/or disability after 10 years (Table S3).

There was no association between annual percentage change in BMD measurements and odds of developing high-intensity pain and/or high disability (Table S4).

Although there was an association between BMD measurements and odds of developing high-intensity pain and/or high disability in older men, over 60 years, there was no association in younger men, based on depression, mobility, and education (Table S5). The prevalence of artefacts was also significantly higher in the older population *>*60 years (data not shown). However, no significant interactions between groups based on age, depression, education, and mobility were found.

The relationship between lumbar BMD at L2–L4 and back pain in those with and without any artefact was examined. In those with any artefact, higher BMD at the lumbar spine was associated with increased incident high-intensity back pain and/or high disability (odds ratio, 1.03; 95% CI, 1.00-1.06) (Table S6). On further exploration excluding those with fractures at baseline, there was no association between lumbar BMD and incident high-intensity back pain and/or high disability. There were too few participants with fracture at baseline to examine the relationship in this subgroup. However, the relationship between lumbar BMD and back pain in those with and without structural change was examined; in those with spinal structural change, increased lumbar spine BMD was not associated with back pain in those with or without structural change. There was no evidence of interaction (*p* = .18).

## Discussion

This is the first population-based longitudinal study in a cohort of men examining the relationship between BMD and the odds of developing high-intensity pain and/or high disability over 10 years. It directly addresses the common community misconception that reduced BMD is associated with back pain, in contrast to the clinician-held belief that there is no relationship between reduced BMD and back pain in the absence of fracture. The study found that BMD was not associated with incident high-intensity back pain and/or high disability.^11^^,^^12^

There is a limited body of literature examining the relationship between BMD and incident back pain in a community-based population, recruited without taking BMD or back pain status into consideration. To our knowledge, there is a single longitudinal study examining this question. It was performed in a birth cohort who had BMD measured at the lumbar spine (L2–L4) at 36 years of age (inception of the study), but back pain was not assessed at that time. Back pain status was assessed 6 years later. In that study, men, but not women, with BMD in the lowest quartile were at increased odds of having back pain 6 years later.^24^ Low BMD at baseline in that population may have been related to existing back pain, but this was not assessed at baseline. Cross-sectional studies have shown an association between low BMD and back pain, which is stronger with longer duration of back pain.^39^ Thus, the association reported by Hoozeman et al^24^ may have been due to low BMD linked to disuse related to back pain. In contrast, the current study examined population-based men across a wide age range (24–87 years), who did not have high-intensity pain and/or high disability at baseline, and followed them prospectively over 10 years for incident high-intensity pain and/or high disability.

The strengths of this study include that this is a population-based study over 10 years, with a population that is representative of the Australian population. In addition, the current study used validated and recommended measurement tools to assess back pain (GCPS) and BMD (DXA).^28^^,^^40^ Those with high-intensity pain and/or high disability at baseline were excluded to limit the possibility of reverse causality. Adjustment was made for spinal artefacts, which may artificially elevate lumbar BMD. In separate analysis excluding men with artefacts, men with fractures, and men with structural changes, there was no association between BMD measures and incident high-intensity pain and/or disability in those without artefacts or fracture or structural changes (Table S6). These results strengthen the conclusion that low BMD is not an independent risk factor for back pain or disability in men.

The study limitations include the low number of men with osteoporosis at any site (L2–L4 = 0.5%, and TH = 0.8%) and fracture in imaging (1.9%) in the study population. To address this, men with osteoporosis and osteopenia at any site were combined: there was no association between osteopenia or osteoporosis and developing high-intensity pain and/or high disability. To more completely explore the association between BMD and incident high-intensity pain and/or high disability, future population-based longitudinal studies could include participants with a wider range of BMD, including those with lower BMD. The follow-up rate was 63.2%, which may have introduced selection bias, as those lost to follow-up were older, more depressed, did not complete secondary school, had lower mobility, and were more likely to have artefacts and lower BMD at the lumbar region. It is possible that our results may underestimate the relationship between BMD and back pain as those men lost to follow-up had characteristics more likely to be associated with incident pain, and had lower BMD. However, these results are generalizable to community-based healthy men. Other forms of bone disease, such as osteomalacia, may cause back pain. Although data regarding the presence of bone disease were not available at baseline, as these are rare in Australia,^41^ this is unlikely to have affected the results. Data regarding incident vertebral fracture at follow-up were not available. Although this may have affected the results, as it is possible that incident vertebral fracture may contribute to developing back pain, we found no significant association. Thus, this limitation is unlikely to have affected the results. As in all observational studies, there is always the potential of residual confounding. Although other variables, such as measures of body composition and socioeconomic status, have been linked to back pain and BMD, these variables are not strongly and consistently related to back pain and BMD, and thus were not included in the analyses. As there may be gender differences, this relationship also needs to be examined in women. There is the possibility of misclassification of back pain intensity and/or disability status as we have measures only at baseline and at 10 years. Although low-intensity pain tends to fluctuate, once high-intensity pain is present, it tends to persist,^42^ suggesting misclassification is less likely.

### Conclusion

This is the first study to suggest longitudinally that BMD is not associated with odds of incident high-intensity back pain or disability in a community-based population of men over 10 years of follow-up. It provides evidence that BMD is not associated with incident back pain and/or disability (incident or deteriorating) in the absence of vertebral fracture. This provides evidence to support health professionals in correcting patients’ beliefs that low BMD (bone health) is related to back pain.

## Supplementary Material

Supplementary_Tables_JBMRPLUS-12-1206_R1_ziae076

## Data Availability

Data availability for Geelong Osteoporosis Study might be available upon reasonable request.
