# Supplementary material for: Bone mineral density is not associated with incident high-intensity back pain: a 10-year cohort study in men
Source: JBMR Plus. 2024 Jun 10;8(8):ziae076. doi: 10.1093/jbmrpl/ziae076 (PMC11234898; doi:10.1093/jbmrpl/ziae076)
Supplement: Supplementary_Tables_JBMRPLUS-12-1206_R1_ziae076 [file supplementary_tables_jbmrplus-12-1206_r1_ziae076.docx]

**Supplementary Table 1: Difference between men in the initial back pain cohort who completed our study (study population) and those who did not (lost-to-follow-up, LTFU) group:**

| **Variables** | **Study population^a^,**  **n= 429** | **LTFU^b^,**  **n= 250** | **p** |
| --- | --- | --- | --- |
| Age^c^ (y) | 54.3 (14.0) | 66.4 (18.5) | <0.001 |
| Body mass index ^c^ (kg/m^2^) | 27.2 (3.8) | 27.3 (4.2) | 0.76 |
| Depression^c^ (total score, 0-21) | 2.5 (2.9) | 3.3 (2.3) | <0.001 |
| Not completed secondary school^d^ | 169 (40.1%) | 128 (52%) | 0.003 |
| Low mobility^d^ | 82 (19.2%) | 83 (33.3%) | <0.001 |
| Artefact^d^ | 200 (47.1%) | 153 (62.4%) | <0.001 |
| Fracture^d^ | 8 (1.9%) | 3 (1.2%) | 0.52 |
| Structural changes^d,e^ | 181 (42.6%) | 140 (57.1%) | <0.001 |
| Other artefacts^d^ | 33 (7.8%) | 28 (11.4%) | 0.11 |
| Lumbar spine | | | |
| Osteoporosis^d^ | 2 (0.5%) | 6 (2.4%) | 0.07 |
| Osteopenia^d^ | 87 (20.3%) | 54 (21.7%) |  |
| BMD^c^, (gm/cm^2^) | 1.28 (0.17) | 1.31 (0.23) | 0.11 |
| Total hip | | | |
| Osteoporosis^d^ | 3 (0.7%) | 7 (3%) | 0.07 |
| Osteopenia^d^ | 132 (31.3%) | 73 (31.7%) |  |
| BMD^c^, (gm/cm^2^) | 1.08 (0.13) | 1.05 (0.14) | 0.01 |

^a^ Study population: participants who completed the chronic pain grade questionnaire at baseline (2006-2010) and follow-up (2016-2021)

^b^ LTFU: Participants who completed the chronic pain grade questionnaire at baseline (2006-2010) but not at follow-up (2016-2021)

^c^Data presented as mean (standard deviation); comparison p-value for Independent T-test

^d^Data presented as number (percentage); comparison p value for Chi-square Test

^e^Structural and degenerative changes includes end plate sclerosis, osteophytes, spondylitis, scoliosis and surgery

BMD, Bone mineral density. Depression (total score)

**Supplementary Table 2: Difference in characteristics of study participants, in those who had persistent no or low-intensity pain and no or low disability and those who developed high-intensity pain and/or high-disability**

| **Variables** | **Pain and/or disability^1^** | | | **Pain^2^** | | | **Disability^1^** | | |
| --- | --- | --- | --- | --- | --- | --- | --- | --- | --- |
|  | No or low-intensity pain and/or disability  n = 392 | High-intensity pain and/or disability  n = 37 | p | No or low pain  n = 398 | High-intensity pain  n = 33 | P | No or low disability  n = 415 | High disability  n = 14 | P |
| Age^3^, years | -2.1 (-6.8, 2.6) | | 0.38 | -1.9 (-7.0, 3.1) | | 0.44 | -1.6 (-9.1, 5.9) | | 0.68 |
| BMI^3^, kg/m^2^ | 0.6 (-0.7, 1.9) | | 0.33 | 0.4 (-1.0, 1.7) | | 0.60 | -0.0 (-2.1, 2.0) | | 1.0 |
| Depressive features | -1.4 (-2.4, -0.4) | | 0.01 | -1.3 (-2.1, -0.5) | | 0.02 | -2.2 (-3.9, -0.5) | | 0.02 |
| BMD^3^, (gm/cm^2^) | | | | | | | | | |
| Lumbar spine | -0.04 (-0.1, 0.02) | | 0.17 | -0.04 (-0.1, 0.02) | | 0.21 | -0.07 (-0.16, 0.02) | | 0.15 |
| Total hip | 0.01 (-0.03, 0.06) | | 0.57 | 0.01 (-0.04, 0.06) | | 0.66 | 0.02 (-0.05, 0.09) | | 0.61 |

^1^Data available for 429 participants who provided pain, disability and BMD data at both time points.

^2^Data available for 431 for participants who provided pain and BMD data, but not disability data at both time points.

^3^Data presented as mean difference (95% Confidence Interval); comparison p-value for Independent T-test

BMI Body mass index; BMD, Bone mineral density

**Supplementary Table 3: Association of BMD measurements and incident pain and/or disability in participants without any pain and/or disability, or between BMD measurements and developing high-intensity pain and/or disability from low-intensity symptoms in participants with low pain and /or low disability at baseline**

|  | **Among those without pain and/or disability at baseline** | | **Among those with low intensity-pain and/or low disability at baseline** | |
| --- | --- | --- | --- | --- |
|  | Total Population 140  Incident high intensity 74 | | Total population 289  Incident high intensity 30 | |
| BMD (g/cm^2^) | Unadjusted  OR (95% CI)^1^ | Adjusted^2^  OR (95% CI)^1^ | Unadjusted  OR (95% CI)^1^ | Adjusted^2^  OR (95% CI) |
| Lumbar spine | 1.0  (0.98-1.02) | 0.99  (0.97-1.01) | 1.01  (0.99-1.03) | 1.00  (0.98-1.03) |
| Total hip | 1.0  (0.98-1.02) | 0.99  (0.96-1.02) | 0.99  (0.96-1.02) | 0.99  (0.95-1.02) |

^1^Odds ratio (95% confidence interval)

^2^Adjusted for age, BMI, depression, mobility (low-mobility), education (Not completed secondary school or lower), smoking (not-current smoker), any artefact (present) and time between two measurements (between 2006-2010 and 2016-2021) (in years)

**Supplementary Table 4a – Annual percentage change in BMD at the lumbar spine and total hip in those with and without incident back pain and/or disability at follow up**

|  | **Pain and/or disability** | | | **Pain** | | | **Disability** | | |
| --- | --- | --- | --- | --- | --- | --- | --- | --- | --- |
|  | No or low-intensity pain and/or disability  n = 392 | High-intensity pain and/or disability  n = 37 | p | No or low pain  n = 398 | High-intensity pain  n = 33 | P | No or low disability  n = 415 | High disability  n = 14 | P |
| Lumbar | | | | | | | | | |
| Absolute change over 10 years | -0.04 (0.08) | -0.05 (0.09) | 0.27 | -0.04  (0.08) | -0.06  (0.09) | 0.21 | -0.04  (0.08) | 0.04  (0.06) | 0.93 |
| Percentage change per year | -0.34% (0.72) | -0.48% (0.79) | 0.29 | -0.34%  (0.72) | -0.50% (0.82) | 0.23 | -0.35%  (0.73) | -0.34%  (0.49) | 0.95 |
| Total hip | | | | | | | | | |
| Absolute change over 10 years | 0.03 (0.05) | 0.02 (0.05) | 0.38 | 0.03  (0.05) | 0.02  (0.05) | 0.34 | 0.03  (0.05) | 0.03 (0.02) | 0.72 |
| Percentage change per year | 0.31% (0.56) | 0.26% (0.48) | 0.59 | 0.32%  (0.56) | 0.25%  (0.50) | 0.53 | 0.31%  (0.56) | 0.41  (0.31) | 0.51 |

Data presented as mean (standard deviation); comparison p-value for Independent T-test

**Supplementary Table 4b: Association of annual percentage change in BMD with developing high-intensity pain and/or high-disability**

| **Change score** | **Pain and disability** | | **Pain** | | **Disability** | |
| --- | --- | --- | --- | --- | --- | --- |
| **BMD (g/cm^2^)** | **Unadjusted**  **OR**  **(95% CI)^1^** | **Adjusted^2^**  **OR**  **(95% CI)^1^** | **Unadjusted**  **OR**  **(95% CI)^1^** | **Adjusted^2^**  **OR**  **(95% CI)^1^** | **Unadjusted**  **OR**  **(95% CI)^1^** | **Adjusted^2^**  **OR**  **(95% CI)^1^** |
| Lumbar spine | 0.77  (0.45-1.24) | 0.80  (0.47-1.35) | 0.74  (0.45-1.21) | 0.76  (0.43-1.34) | 1.03  (0.48-2.20) | 1.14  (0.49-2.66) |
| Total hip | 0.84  (0.43-1.61) | 0.89  (0.46-1.72) | 0.80  (0.40-1.61) | 0.87  (0.42-1.78) | 1.38  (0.53-3.61) | 1.52  (0.60-3.67) |
| **BMD (g/cm^2^)** | **Unadjusted**  **OR**  **(95% CI)^1^** | **Adjusted^3^**  **OR**  **(95% CI)^1^** | **Unadjusted**  **OR**  **(95% CI)^1^** | **Adjusted^3^**  **OR**  **(95% CI)^1^** | **Unadjusted**  **OR**  **(95% CI)^1^** | **Adjusted^3^**  **OR**  **(95% CI)^1^** |
| Lumbar spine | 0.77  (0.45-1.24) | 0.77  (0.45-1.32) | 0.74  (0.45-1.21) | 0.74  (0.42-1.31) | 1.03  (0.48-2.20) | 1.12  (0.47-2.65) |
| Total hip | 0.84  (0.43-1.61) | 0.88  (0.46-1.71) | 0.80  (0.40-1.61) | 0.86  (0.42-1.75) | 1.38  (0.53-3.61) | 1.47  (0.58-3.69) |

^1^Odds ratio (95% confidence interval)

^2^Adjusted for age, BMI, depression, mobility (low-mobility), education (Not completed secondary school or lower), smoking (not-current smoker), any artefact (present) and time between two measurements (between 2006-2010 and 2016-2021) (in years)

^3^Adjusted for age, BMI, depression, mobility (low-mobility), education (Not completed secondary school or lower), smoking (not-current smoker), any artefact (present), time between two measurements (between 2006-2010 and 2016-2021) (in years) and baseline BMD

BMD=bone mineral density.

**Supplementary Table 5:** **Association of bone mineral density, incidence of high-intensity pain and/or high disability based on age, depression, mobility, education and fracture categories**

| **Age (median age 60 years)** | | | | | |
| --- | --- | --- | --- | --- | --- |
| **BMD (g/cm^2^)** | **Univariate analysis**  OR (95%CI)^1^ | | **Multivariate analysis^2^**  OR (95%CI)^1^ | | P^3^ |
|  | <60 years  No-low=248  High=24 | >60 years  No-low=144  High=13 | <60 years  No-low=248  High=24 | >60 years  No-low=144  High=13 |  |
| Lumbar spine | 1.0  (0.97-1.02) | 1.03  (1.01-1.06) | 1.00  (0.96-1.03) | **1.03**  **(1.00-1.07)** | 0.33 |
| Total hip | 0.98  (0.95-1.02) | 1.01  (0.96-1.06) | 0.99  (0.95-1.03) | 1.02  (0.96-1.07) | 0.80 |
| **Depression (total score 0-21)** | | | | | |
|  | **Univariate analysis**  OR (95%CI)^1^ | | **Multivariate analysis^4^**  OR (95%CI)^1^ | | P^3^ |
|  | <8  No-low=378  High=33 | >8  No-low=12  High=4 | <8  No-low=378  High=33 | >8  No-low=12  High=4 |  |
| Lumbar spine | 1.01  (0.99-1.03) | 1.01  (0.96-1.07) | 1.02  (1.0- 1.04) | - | - |
| Total hip | 1.00  (0.98-1.03) | 0.85  (0.71-1.01) | 1.01  (0.98-1.04) | - | - |
| **Mobility** | | | | | |
|  | **Univariate analysis**  OR (95%CI)^1^ | | **Multivariate analysis^5^**  OR (95%CI)^1^ | | P^3^ |
|  | High-mobility  No-low= 321  High=25 | Low-mobility  No-low=70  High= 12 | High-mobility  No-low= 321  High=25 | Low-mobility  No-low=70  High= 12 |  |
| Lumbar spine | 1.01  (0.98, 1.03) | 1.02  (0.99, 1.05) | 1.01  (0.98-1.04) | 1.02  (0.98-1.06) | 0.42 |
| Total hip | 1.00  (0.97, 1.03) | 0.97  (0.92, 1.03) | 1.00  (0.97-1.04) | 0.98  (0.91-1.05) | 0.55 |
| **Education** | | | | | |
|  | **Univariate analysis**  OR (95%CI)^1^ | | **Multivariate analysis^6^**  OR (95%CI)^1^ | | P^3^ |
|  | Not completed  No-low=153  High= 16 | Completed  No-low=231  High=21 | Not completed  No-low=153  High= 16 | Completed  No-low=231  High=21 |  |
| Lumbar spine | 1.02  (0.99-1.05) | 1.01  (0.98-1.03) | 1.01  (0.98-1.04) | 1.01  (0.99-1.04) | 0.89 |
| Total hip | 0.98  (0.94-1.02) | 1.01  (0.97-1.04) | 0.97  (0.92-1.02) | 1.01  (0.98-1.05) | 0.16 |

^1^Odds ratio and (95% Confidence Interval).

^2^adjusted for low mobility, not completed secondary school or lower, depression, not-current smoker, BMI, any artefact and time between two measurements (between 2006-2010 and 2016-2021) (in years)

^3^Interaction between groups

^4^adjusted for low mobility, completed secondary school or lower, age, not-current smoker, BMI, any artefact and time between two measurements (between 2006-2010 and 2016-2021) (in years)

^5^adjusted for not-completed secondary school or lower, age, not-current smoker, BMI, depression, any artefact and time between two measurements (between 2006-2010 and 2016-2021) (in years)

^6^adjusted for low mobility, age, not-current smoker, BMI, depression, any artefact and time between two measurements (between 2006-2010 and 2016-2021) (in years)

**Supplementary Table 6: Association of measures of BMD with developing high-intensity pain or high-disability based on artefacts**

| **BMD (g/cm^2^)** | **High intensity pain and/or disability**  Odds ratio (95% confidence interval) | | **P for interaction** |
| --- | --- | --- | --- |
| **Artefact** | | | |
|  | **Present** | **Absent** |  |
| Lumbar spine**^1^** | 1.03  (1.00-1.06) | 0.99  (0.96-1.02) | 0.04 |
| **Fracture** | | | |
|  | **Present** | **Absent** |  |
| Lumbar spine**^2^** | - | 1.01  (0.99-1.03) | - |
| **Structural changes** | | | |
|  | **Present** | **Absent** |  |
| Lumbar spine**^3^** | 1.03  (1.00-1.06) | 0.99  (0.96-1.03) | 0.18 |

^1^Adjusted for age, BMI, depression, mobility (low-mobility), education (Not completed secondary school or lower), smoking (not-current smoker) and time between two measurements (between 2006-2010 and 2016-2021) (in years)

^2^Adjusted for age, BMI, depression, mobility (low-mobility), education (Not completed secondary school or lower), smoking (not-current smoker), other spinal abnormality except fracture (present) and time between two measurements (between 2006-2010 and 2016-2021) (in years)

^3^Adjusted for age, BMI, depression, mobility (low-mobility), education (Not completed secondary school or lower), smoking (not-current smoker), other spinal abnormality except structural changes (present) and time between two measurements (between 2006-2010 and 2016-2021) (in years)
